# Lectures on Diseases of the Skin

**Published:** 1886-04

**Authors:** Henry Wile

**Affiliations:** Lecturer on Dermatology, Atlanta, Ga.


THE ATLANTA MEDICAL AND SURGICAL JOURNAL.

VOL. III. APRIL, 1886. No. 2.

### Original Communications.

### LECTURES ON DISEASES OF THE SKIN.

DELIVERED AT THE ATLANTA MEDICAL COLLEGE DURING THE SESSION OF 1885–6. BY HENRY WILE, M. D., LECTURER ON DERMATOLOGY, ATLANTA, GA.

### LECTURE II.

ANATOMY CONTINUED—SEBACEOUS GLANDS—HAIR—HAIR FOLLICLE—NAIL—PHYSIOLOGY—FUNCTION OF PROTECTION—SECRETION AND EXCRETION—RESPIRATION—ABSORPTION—SENSATION.

Stenographically Reported for THE ATLANTA MEDICAL AND SURGICAL JOURNAL by W. Kay Tewksbury.

The sebaceous glands are indentations of the epidermis, containing, therefore, in their structure all the epidermal layers, especially those of the rete mucosum. They are situated in the upper layers of the corium, and are found universally distributed, except in the palms of the hands, soles of the feet, backs of the terminal phalanges and glans penis. They are simple or racemose glands and vary in size, being smaller on the scalp, larger on the nose, at the angles of the mouth and in the genital region. They open by means of a duct, as a rule, into the hair follicle, being then appendages of the hair. Others open directly upon the surface o the skin and have a rudimentary or lanugo hair as an appendage. A few, as around the nipple, open directly upon the surface without any relations to hair. The ceruminous glands in the external auditory meatus and the Meibomian glands of the eyelids are also classed as true sebaceous glands. The duct is made up of one or more layers of flattened or cuboidal epithelial cells, and descending it communicates with the body of the gland which is formed of one or more acini. The acini are composed of cuboidal epithelial cells situated upon a basement membrane. These cells are granular, in a state of fatty degeneration, and are constantly being thrown off from the basement membrane, so that the body of the gland is filled with these cells, which, as they reach the hair follicle, become converted into fatty particles called *sebum*.

The hairs are also epidermoidal structures, each fitting into an invagination of the skin called the hair follicle. They vary in length, shape and color according to location, whether in the same or different individuals; also according to age. The part above the surface of the skin is called the shaft; that below the surface, the root. The hair is universal in its distribution, and is found everywhere, except upon the eyelids, lips, palms and soles, extensor surfaces of the terminal phalanges, the inner surface of the prepuce and the glans penis. There are three varieties: first, long hair as on the scalp; second, short, coarse hair as the eyebrows and lashes; third, fine lanugo hair as on the face and trunk. The root of the hair extends to the lower layers of the corium and ends in an expansion called the bulb, which is hollow and embraces the papilla that rises from the base of the follicle much after the manner of a ball and socket joint. The hair is composed of a cortical and medullary substance, and is surrounded by a delicate, cellular membrane called the cuticle, which presents on microscopical examination of its surface a serrated appearance. The cortical substance, which forms the bulk of the hair, taking up three-fourths of its diameter and giving it form and stability, is composed of flattened, spindle-form, horny plates or filaments closely cemented together, but which can easily be isolated for examination by treating the hair with concentrated sulphuric acid. These horny plates contain granular matter or pigment which determines the color of the hair. This pigment is absent in gray hair. As the papilla is approached, the cells of the cortical substance assume the character of the cells of the rete mucosum, becoming more ovoidal, cuboidal and finally cylindrical in form. The medullary substance forming the remaining fourth of the hair diameter occupies the centre, and is composed of granular, polyhedral, epithelial cells. The cells contain granules of fat, and, according to some authorities, air bubbles are occasionally seen between the cells. In lanugo hairs the medullary substance is wanting.

The hair follicle extends more or less obliquely into the corium, its depth varying according to the length of the hair which it contains. We distinguish the neck of the follicle as that part where the sebaceous gland has its outlet. The hair follicle is composed of connective tissue layers and cellular layers. Of the former there are three layers: first, the external fibrous layer, which is a continuation of the reticular layer of the corium, composed of bundles of connective tissue running parallel with the root of the hair; second, the middle fibrous layer, a continuation of the papillary layer of the corium and composed of delicate bundles of connective tissue fibre arranged transversely to the long axis of the hair; and third, the internal or vitreous layer, an almost transparent, homogeneous membrane, most apparent in transverse sections of the follicle. According to one authority, the last is a condensation of the middle fibrous layer inwardly, nitrate of silver staining showing a delicate fibrillar structure.

The hair papilla is a conical structure projecting from the base of the follicle and is a continuation of the middle and internal layers of the follicle. It has the same structure as a papilla of the corium, and indeed may be regarded as a modified papilla. In it we distinguish the neck or constricted portion at the base and the body or upper expanded portion. Each papilla contains a loop of blood-vessels, also a non-medullated nerve.

The cellular layers of the hair follicle, also called the root-sheaths, are a continuation of the epidermal layers and are divided into external and internal root-sheath. The external root-sheath, or that next to the vitreous layer, is a continuation of the rete malpighii, and is composed of several layers of cuboidal epithelial cells, which taper off into a single row of cells as the papilla is reached. The internal root-sheath is a continuation of the horny layers of the epidermis, which extends into the follicle and as far as the neck retains all its characteristics; but below that point the component cells are changed. Here two layers are to be distinguished. The outer, also called the layer of Henle, consists of one layer of flattened non-nucleated more or less elongated epithelial cells, which at the lower part of the follicle appear as a single row of scales. The inner layer, or layer of Huxley, is composed of a double layer of flattened fusiform cells, containing nuclei at the lower part of the follicle, but growing more and more homogeneous as the neck of the follicle is approached. At that point it unites with the layer of Henle, forming a single layer. Immediately below the hair follicle, and acting, as it were, as a connecting band between the hair follicle and subcutaneous connective tissue, is a structure called the *columnæ adiposæ*, composed of strands of loose areolar tissue and numerous fat cells.

An interesting fact connected with the hair is that it is the last structure of the human body to decompose.

The nail, a more or less oblong, flattened or convex body, occupying the dorsal surface of the terminal phalanges, is, like the hair, an appendage of the skin. The part which is visible is called the body. The part which extends under the cover of the skin and is hidden from view is called the root. The surface of the cutis covered by the body is called the bed of the nail and that part sustaining the root is called the matrix. The substance of the nail is modified epidermis made up of thin horny epithelial plates, which are closely cemented together. It is thinnest at the root and acquires thickness as it advances towards the free edge. The bed of the nail consists of ridges and grooves which are covered entirely with *prickle cells*. Between the body and the root of the nail is a crescentic spot lighter in color than the body and called the *lunula*. This lighter color is explained by the fact that this part is less transparent than other portions of the nail, and it is due to the presence of opaque cells which reflect the light and do not allow the blood to shine through. The condition is analogous to that produced by a blow on the nail which causes an opacity of the cells that is indicated by a white spot. It requires about five months for the nail to grow its entire length.

### PHYSIOLOGY.

The physiological relations which the skin sustains to the rest of the organism are three-fold. First, on account of its position and peculiar anatomy, the skin serves as a protection and support to more delicate subjacent structures. Second, by means of its glandular apparatus it acts as a medium of secretion and excretion. Third, through the delicate nerve terminals it affords a means of communication with the external world, being the seat of the sense of touch.

The epiderm, especially the horny layer, is devoid of a sensory apparatus and therefore protects the organism from the appreciation of minor sensation, or at least diminishes the appreciation of painful impressions from without. In addition, on account of the resistant character of the epidermal tissue, it resists to a greater or less degree the action of corroding liquids. Another important protective function of the epiderm is the conservation of animal heat. Being a poor conductor of heat it prevents excessive radiation, and to it in a measure may be attributed the maintenance of a comparatively uniform body temperature. It also holds back the juices which circulate in the lower cutaneous tissues, for if the epidermal cells are removed there will be an oozing of serum which continues until they are restored.

The hair acts as an aid to the epidermis in preventing a too rapid loss of heat by radiation, thus forming a protection against atmospheric temperatures lower than that of the blood. The nail, which is a body corresponding to the claw in the lower animals, serves as a shield to the ends of the fingers and toes. Its other functions are mainly prehensile. The corium on account of its elasticity is extensible and forms a frame-work and support for the glandular, vascular and nervous cutaneous structures. The protective function of the subcutaneous fat layer deserves specia mention, as it acts like a cushion in receiving any sudden injuries, such as blows from without.

The excretory and secretory functions are carried on by the sebaceous and sweat glands. As a rule the sebaceous glands are appendages of the hair and secrete a fatty or oily substance which lubricates the hair and surface of the skin, imparting a lustre to the former and keeping the skin soft, also protecting parts from injury by friction as about the joints. This secretion is formed by a fatty degeneration of the cells lining the glands. It passes along he duct to the hair follicle, and thence to the surface of the skin, its passage being facilitated by the contractions of the *arrector pili* muscle which presses the gland against the hair follicle. In the external auditory canal these glands, known as the ceruminous glands, secrete a fatty substance of some consistence, known as the ear-wax, which protects the tympanum of the ear and surrounding epiderm and is poisonous to little insects that may invade this region. On the edge of the eyelids the Meibomian glands secrete an oily material which acts as a guard to the tears, preventing them from running over the free edges of the conjunctiva on to the cheeks.

The sweat glands, situated in the subcutaneous connective tissue, secrete from the arterial blood, that circulates in a complicated plexus around each glandular coil, a thin watery fluid by means of the epithelial cells lining the glands. The secretion when fresh has a specific gravity of about 1004, an acid reaction, and contains about ninety-nine per cent. of water and one per cent. of solid constituent, of which the chief ingredient is sodium chloride. The acid reaction is due to the presence of a volatile acid principle which also lends to the fluid a characteristic odor.

The secretion goes on continually. When slight it is known as insensible perspiration, and when noticeable it is called sensible perspiration. The quantity of the secretion depends upon the temperature of the surrounding atmosphere, and also upon the action of the heart or blood pressure whereby the cutaneous blood-vessels become more or less congested. These blood-vessels are especially under the influence of the vaso-motor nerves, the excitation of which, through emotions of various kinds, as fright, anxiety, intense pain, may cause normally an excessive secretion. The sweat glands are surrounded by fibres of smooth muscular tissue, by means of which the glands are enabled to discharge their contents upon the surface.

The perspiratory apparatus has a double function, acting at once as an emunctory, whereby effete material is eliminated, and as a regulator of body temperature. Under the latter, when the body temperature is raised in consequence of a rapid internal heat generation or atmospheric temperature above 100° F., it is soon brought to the normal standard by a great activity of the sweat glands. A copious secretion is poured out on the surface of the skin, and by rapid evaporation, in which a liquid passes to a gaseous state, heat is rendered latent, and a lowering of temperature is effected.

The respiratory function, corresponding to that of the lungs, is also discharged by the skin, oxygen being taken up and carbonic acid gas exhaled. This has been shown by the experiment of placing a limb in an air-tight chamber and subsequently making an analysis of the air in the chamber. This analysis showed a loss of oxygen and a gain in carbonic acid gas.

The process of absorption is carried on mainly by means of the glandular apparatus; also to some extent by the cells of the epidermis or the intercellular spaces. The skin is impervious to fluids and finely powdered substances when these are merely brought in contact with the intact epidermis, though if the surface is abraded absorption may occur. It readily takes place when the fluid or finely divided solid particles are brushed or rubbed into the skin. Thus oils and ointments containing solid particles may be rubbed or pressed on the surface of the skin and brought mechanically in contact with the lymphatic system. Neumann, of Vienna, has shown, by sections of skin that had been subjected to repeated inunctions of mercurial ointment, the presence of particles of the metal in the glands and lymphatics.

The skin, as an organ of sense, discharges a most important function. It is the seat of terminal sensory nerves, which end in the so-called touch or tactile corpuscles situated in the papilla of the corium. The sense of touch varies according to location, and is most acute on the tips of the fingers and toes and about the lips. In these places we find the greatest number of tactile corpuscles. That the variation in the acuteness of this sense is due the number of tactile corpuscles present is shown by the æsthesiometer. On the flexor surface of the terminal phalanges the points are recognized at one-tenth of an inch, on the second phalanges two-tenths, on the extensor surface of second phalanges four-tenths, on the sternum two inches, on the back three and one-half inches. Some physiologists make a functional division of the sensory cutaneous nerves into those filaments appreciating painful sensation, and those receiving impressions of resistance or pressure and temperature changes. Though such a division is at present purely arbitrary, I think there is much in favor of the idea that the sensory nerves of the skin possess in a varying degree the power of receiving impressions, and that some filaments appreciate more delicate impressions than others.

